# World Heart Federation Roadmap for Hypertension – A 2021 Update

**DOI:** 10.5334/gh.1066

**Published:** 2021-09-10

**Authors:** Panniyammakal Jeemon, Tania Séverin, Celso Amodeo, Dina Balabanova, Norm R. C. Campbell, Dan Gaita, Kazuomi Kario, Taskeen Khan, Rita Melifonwu, Andrew Moran, Elijah Ogola, Pedro Ordunez, Pablo Perel, Daniel Piñeiro, Fausto J. Pinto, Aletta E. Schutte, Fernando Stuardo Wyss, Lijing L. Yan, Neil R. Poulter, Dorairaj Prabhakaran

**Affiliations:** 1 Sree Chitra Tirunal Institute for Medical Sciences and Technology, Trivandum, IN; 2 World Heart Federation, Geneva, CH; 3 Universidade Federal de São Paulo (UNIFESP), São Paulo, BR; 4 London School of Hygiene & Tropical Medicine, London, GB; 5 Libin Cardiovascular Institute of Alberta, University of Calgary, CA; 6 Universitatea de Medicina si Farmacie Victor Babes, Timisoara, RO; 7 Jichi Medical University School of Medicine, Shimotsuke, Tochigi, JP; 8 University of Pretoria, ZA; 9 Stroke Action Nigeria, Onitsha, Anambra State, NG; 10 Columbia University and Resolve to Save Lives, New York, US; 11 University of Nairobi, KE; 12 Pan American Health Organization, Washington, DC, US; 13 London School of Hygiene & Tropical Medicine and World Heart Federation, Geneva, GB; 14 Universidad de Buenos Aires, AR; 15 Santa Maria University Hospital (CHULN), CAML, CCUL, Lisboa, PT; 16 University of New South Wales; The George Institute for Global Health, Sydney, AU; 17 Cardiovascular Technology and Services of Guatemala – CARDIOSOLUTIONS, Guatemala, GT; 18 Duke Kunshan University, Kunshan, CN; 19 Imperial College London, London, GB; 20 Public Health Foundation of India, Gurugram, IN

**Keywords:** World Heart Federation, WHF, hypertension, raised blood pressure, raised BP, policy

## Abstract

The World Heart Federation (WHF) Roadmap series covers a large range of cardiovascular conditions. These Roadmaps identify potential roadblocks and their solutions to improve the prevention, detection and management of cardiovascular diseases and provide a generic global framework available for local adaptation.

A first Roadmap on raised blood pressure was published in 2015. Since then, advances in hypertension have included the publication of new clinical guidelines (AHA/ACC; ESC; ESH/ISH); the launch of the WHO Global HEARTS Initiative in 2016 and the associated Resolve to Save Lives (RTSL) initiative in 2017; the inclusion of single-pill combinations on the WHO Essential Medicines’ list as well as various advances in technology, in particular telemedicine and mobile health. Given the substantial benefit accrued from effective interventions in the management of hypertension and their potential for scalability in low and middle-income countries (LMICs), the WHF has now revisited and updated the ‘Roadmap for raised BP’ as ‘Roadmap for hypertension’ by incorporating new developments in science and policy. Even though cost-effective lifestyle and medical interventions to prevent and manage hypertension exist, uptake is still low, particularly in resource-poor areas. This Roadmap examined the roadblocks pertaining to both the demand side (demographic and socio-economic factors, knowledge and beliefs, social relations, norms, and traditions) and the supply side (health systems resources and processes) along the patient pathway to propose a range of possible solutions to overcoming them. Those include the development of population-wide prevention and control programmes; the implementation of opportunistic screening and of out-of-office blood pressure measurements; the strengthening of primary care and a greater focus on task sharing and team-based care; the delivery of people-centred care and stronger patient and carer education; and the facilitation of adherence to treatment. All of the above are dependent upon the availability and effective distribution of good quality, evidence-based, inexpensive BP-lowering agents.

## Background

The World Heart Federation (WHF) has produced a series of Roadmaps over the past years as part of its commitment to improve global cardiovascular health. In line with the organisation’s mission to translate science into policy and to stimulate and catalyse the exchange of information, ideas, and practices across borders, WHF Roadmaps identify potential roadblocks and their solutions on the pathway to effective prevention, detection, and management of cardiovascular disease (CVD) and guide priority interventions on a global level. Roadmaps focusing on raised blood pressure (BP) [1], secondary prevention and tobacco control [2], atrial fibrillation [3], cholesterol [4], rheumatic heart disease [5], CVD & diabetes [6], heart failure [7], and Chagas disease [8], have already been published. Mostly these Roadmaps provide a generic global framework available for local adaptation and are intended to serve as a basis for developing region- or country-specific action plans.

The WHF Roadmaps are aimed to support Goal 3 of the United Nations Sustainable Development Goals (SDGs), ‘Ensure healthy lives and promote well-being for all at all ages’, which includes a target [3,4] of one-third reduction in premature mortality from non-communicable diseases (NCDs) by 2030.

Raised BP, is the leading cause of death globally, claiming approximately 10.8 million lives in 2019 [9]. These lives include not only cardiovascular disease but also cerebrovascular disease. Raised BP is therefore highly relevant to the SDG target on NCDs. Treatment of hypertension has also been shown to be highly cost-effective. Given the substantial benefit accrued from effective interventions in the management of hypertension and the potential for scalability of such interventions in low- and middle-income countries (LMICs), the WHF revisited and updated the ‘Roadmap for raised BP’ as ‘Roadmap for hypertension’ by incorporating new developments in science and policy. The move from a focus on raised BP to hypertension is motivated by pragmatic reasons, aiming for short term gains by focusing on hypertension rather than the whole range of elevated blood pressure. This updated Roadmap on hypertension also incorporates findings and feedback on barriers and enablers of hypertension management from an online consultation of 106 WHF members from 46 different countries.

## A rationale for the update

Globally, hypertension was estimated to be prevalent in 1.39 billion adults in 2010 [10]. Further, based on the revised The Global Burden of Disease study (GBD) estimates in 2019, high systolic blood pressure affects over 4 billion adults [11]. Although the prevalence of raised BP remained stable in high-income countries (HICs) for the last 1–2 decades, it is still increasing in low- and middle-income countries (LMICs) [10,12,13]. Improvements in hypertension awareness, treatment, and control rates have been documented in HICs. However, this progress in HICs seems to have mainly occurred in the 1990s to early-mid 2000s and since then plateaued [14] or deteriorated [15,16]. Despite the availability of effective treatments, hypertension management remains poor and is characterised by low levels of awareness, treatment, and control, especially in LMICs [17].

Beyond the direct health impact of hypertension and the attributable disease burden, there is a strong rationale to tackle hypertension effectively to gain economic development. For example, the global health care savings from effective management of BP alone have been estimated at $100 billion per year [18]. Each dollar spent on clinical management of cardiovascular risks, including hypertension, is estimated to return $3 USD by the NCD Global Business Plan [19]. Therefore, from a health policy perspective, improving the detection, management, and control of hypertension deserves high priority.

The first WHF Roadmap on the management and control of raised BP was published in 2015 [1]. Since then, there have been advances in the field of hypertension, including 1) several new sets of national and international clinical guidelines including those of the AHA/ACC in 2017, the European Society of Cardiology (ESC) and the European Society of Hypertension (ESH) in 2018, and the International Society of Hypertension (ISH) in 2020 [12], 2) the launch of the WHO Global HEARTS Initiative in 2016 [20,21], the launch of MMM screening around the world and the associated Resolve to Save Lives (RTSL) initiative in 2017 to support government and civil society groups in implementing scalable, proven hypertension control policies and clinical implementation strategies [22], 3) the launch of the Sao Paulo call to action [23], 4) the inclusion of single-pill combinations on the WHO Essential Medicines’ list in 2019 [24] 5) advances in technology, in particular telemedicine and mobile health (mHealth) and 6) finally, availability of real world experiences from several WHF members, such as the Pan-African Society of Cardiology (PASCAR), and the Colombian Society of Cardiology and Cardiovascular Surgery (SCCCC) after the local or regional adaptation of the first WHF Roadmap in improving hypertension control (case studies 1–3).

The present Roadmap update builds on the original publication and summarises:

- new evidence on burden of disease, epidemiology, treatment, emerging technologies, health system strategies, and policies that can inform, support, and improve the detection and management of people living with hypertension and
- lessons learnt from the WHF Roadmap use and implementation in different countries and settings.

## Recent trends in hypertension

### Defining our terms

Raised or high BP is a condition described as an elevation in systolic BP beyond the level of exposure that minimizes risk at the population level (110–115 mmHg). Hypertension is a long-term medical condition characterized by a persistent elevation in office/clinic BP (systolic BP ≥140 and/or diastolic BP ≥90 mmHg) [2,25], which necessitates long-term management. Even though the latest AHA/ACC guideline defines hypertension as persistent elevation in office/clinic BP (systolic BP ≥130 and/or diastolic BP ≥80 mmHg (Table 2) [26], for the purpose of this Roadmap, we follow the most commonly used definition of hypertension due to the following pragmatic reasons; (a) well-documented and indisputable benefits of initiating treatment in individuals with systolic BP ≥140 and/or diastolic BP ≥90 mmHg, (b) global acceptance of the definition and the general inertia in initiating treatment in individuals where the benefit has not been unequivocally proven in randomised controlled trials and (c) significant and unrealistic increase in the number of individuals requiring BP management at the primary care level with the AHA/ACC definition of hypertension and the subsequent impact on the strained health system in LMICs with uncertain benefit [27].

### Epidemiology/Burden of disease

According to the latest GBD update, raised BP – defined as systolic BP > 110 to 115 mm Hg, that is, the range that minimizes risk at the population level, affected 4.06 billion adults globally in 2019 [11]. Global hypertension prevalence estimates in 2010 suggest that it affects 1.39 billion adult individuals [10]. Prevalence of hypertension varies across regions and countries. In 2015, 20% of men in the high-income Asia Pacific region were affected, compared to 33% in Central and Eastern Europe. Among women, these rates ranged from 11% in the high-income Asia Pacific region to 28% in sub-Saharan Africa [28]. Two-thirds of individuals with hypertension live in LMICs [10]. The mean systolic BP appears to be increasing over time in east, southeast, and south Asia, Oceania, and Sub-Saharan Africa while decreasing in other regions [23]. Further, BP also increases with age in almost all societies, such that an estimated 90% of adults living to 80 years of age are likely to develop hypertension [29]. Compared to men of a similar age, premenopausal women tend to be less affected. However, after the menopause a steeper increase in hypertension prevalence is observed in women, therefore the age-specific prevalence is higher in women than men over the age of 65 years [30].

The global burden of hypertension is daunting. In 2019, it caused an estimated 10.8 million deaths (approximately 19% of overall deaths) and contributed to 235 million DALYs (9.3% of total DALYs) [9,31]. Hypertension causes over 50% of heart disease, stroke, and heart failure cases [32]. Compared to HIC, in LMICs, heart disease and stroke often occur in younger people in their productive life years [10]. Hypertension causes over 40% of deaths in people with diabetes and is also a leading risk for foetal and maternal death in pregnancy, dementia, renal failure (particularly in people of African ancestry), and blindness [23].

The economic burden of hypertension is also significant: an estimated 10% of global health care spending is directly related to raised BP and its complications such as ischemic heart disease, heart failure, and stroke [18]. This proportion reaches nearly 25% of health care spending in Eastern Europe and Central Asia [18]. Social determinants of health are closely associated with hypertension. Lower socio-economic status (SES) based on indicators such as income, occupation, and education is associated with an increased risk of hypertension. It is therefore particularly important to identify and monitor hypertension to reduce the risk among the most vulnerable groups [33,34]. Dealing with the consequences of hypertension often represents a heavy financial burden for those affected and their families, due to out-of-pocket payments for consultations and medications and to catastrophic costs associated with sequela CVDs (survivors of stroke, heart failure or acute myocardial infarction) and with premature deaths [34,35,36].

Age and family history are well-documented risk factors for hypertension [29]. Lifestyle-related risk factors are also well-identified and include unhealthy diet, particularly high salt content or insufficient fruit and vegetables; harmful use of alcohol; physical inactivity; and overweight and obesity [1]. In addition, a range of environmental factors such as fine particulate matter and extreme weather conditions adversely affect BP and risk of CVD [37,38,39].

### Screening and diagnosis of hypertension

Initial screening for hypertension should take place from the age of 18, and then ideally repeated every two years, depending on the availability of resources. Guidelines have taken a more pragmatic approach of recommending repeat measurement between 3–5 years depending on BP [25]. Opportunistic screening may be ideal for adolescents younger than 18 years of age and with specific risk factors such as use of oral contraception, obesity and family history of hypertension or premature CVD. Poor awareness among adults who have never had a single BP measured especially in LMIC settings [40,41] calls for sustained community and workplace screening programmes for hypertension. Evidence suggests that settings-based screening programmes (screening at workplaces, schools, place of worships, barbershops, etc.) improve the treatment and control rate of hypertension [42]. However, limited data are available on the effectiveness of different screening strategies for hypertension in reducing mortality and morbidity associated with hypertension [43]. The equipment used for screening, human resources involved, and training determine the cost and affordability of screening strategies. A variety of equipment is used for BP measurements and include arm- or wrist-cuff auscultatory (aneroid, mercury, digital) or automated electronic devices, or cuff-less sensor-based devices. Additionally, they use different techniques such as auscultatory, oscillometric, tonometry, and pulse wave record analysis for BP measurement. However, the accuracy of these devices varies widely and reduces the efficacy of mass BP screening programmes to diagnose hypertension accurately. Further, access and procurement of BP machines that have been validated for accuracy remains a challenge in low-resource settings.

All hypertension guidelines recommend identification of persistently high BP for the diagnosis of hypertension. The requirement for multiple measurements necessitates multiple clinic visits or out of clinic BP measurements (home BP monitoring [HBPM]) or ambulatory BP measurements (ABPM). Improved control, enhanced diagnosis of white-coat hypertension, correct identification of masked hypertension, and better prediction of cardiovascular risk are the advantages of out of clinic BP measurements [44]. Further, out of clinic BP measurements facilitate telemonitoring, detect variability, and encourage involvement in hypertension management [25]. Out of clinic BP measurements reduce misdiagnosis, and often the additional costs from such monitoring are offset by cost savings from efficient and targeted treatment [45]. However, availability and accessibility issues limit the use of out of clinic BP measurements in LMIC settings.

#### Non-pharmacological interventions

Non-pharmacological interventions are an important component of hypertension management. Further, hypertension management guidelines in general recommend non-pharmacological interventions for the prevention of hypertension. Non-pharmacological interventions are also recommended along with pharmacological treatment in individuals with hypertension to achieve better control of BP and reduce the dosage of drugs or pill burden. Hypertension management often requires a multi-faceted approach to more than one risk factor and an iterative process that may require continuous and life-long adherence. Reducing cardiovascular risk by targeting multiple CVD risk factors at the same time rather than targeting BP reduction alone is recommended. Recommended non-pharmacological strategies are summarised in Table 1.

**Table 1:** Important lifestyle intervention strategies for the prevention and management of hypertension.

Approach	Recommendations

Weight management	Best goal is ideal body weight. Expect about 1 mmHg for every 1-kg reduction in body weight [131].
DASH Diet	Consume a diet rich in fruits, vegetables, whole grains, and low-fat dairy products, with reduced content of saturated and total fat. Expect up to 11 mmHg reduction in SBP [132,133].
Reduced intake of dietary sodium	Aim for at least a 1000 mg/d reduction per day. (one fifth teaspoon of salt). Expect up to 5 mmHg reduction in SBP [134,135].
Enhanced intake of dietary potassium	Aim for 3500–5000 mg/d per day. Preferably by a diet (such as locally available fruits and vegetables) rich in potassium. Replacing high-sodium salt with potassium-rich salt is also recommended. Expect up to 4 mmHg reduction in SBP [136,137].
Physical activity	Aerobic exercise of 90–150 min/week. Expect up to 5 mmHg reduction in SBP [138]. Dynamic resistance exercise of 90–150 min/week. Expect up to 4 mmHg reduction in SBP [138]. Isometric resistance exercise of three sessions/week. Expect up to 5 mmHg reduction in SBP [139,140]. Ambulatory physical activity such as step count (8000 to 10,000) per day [141].
Moderation in alcohol intake	Complete abstinence or limit alcohol intake to ≤2 standard drinks per day with 2 days off per week. Expect up to 3–4 mmHg reduction in SBP [142].

DASH = The Dietary Approaches to Stop Hypertension, SBP = Systolic Blood Pressure.

Non-pharmacological, non-personal interventions are also important for hypertension prevention and control. Such policy interventions are referred to in Section E – Focus areas, barriers, and enablers to scale up recommendations.

#### Pharmacological interventions

Hypertension guidelines in general support low dose pharmacological therapy initially (albeit of two agents in several guidelines) and subsequent up-titration based on the achieved BP and tolerability. The recommended pharmacological intervention options for the management of hypertension are largely similar despite some variations among different guidelines. Similarly, the latest guidelines are relatively consistent in terms of treatment threshold for initiating antihypertensive agents and the target BP to be achieved during treatment. The most recent guidelines for the management of hypertension are summarised in Table 2.

**Table 2:** Recommendations from recent hypertension management guidelines.

Name	Diagnosis	Target/Threshold	Treatment (Initial)	Treatment (Sequencing)

ISH, Unger et al., 2020 [12]	≥140/90 mm Hg (clinic BP).	Aim for at least a 20/10 mmHg BP reduction, ideally to <140/90 mmHg. Target BP <130/80 mmHg if tolerated and age <65 years (but >120/70 mmHg).	A+C (low dose)	A+C (full dose) A+C+D A+C+D+Spironolactone Treatment. Intensity stratified by CVD risk
NICE, 2019 [143]	≥140/90 mm Hg (clinic BP).	Aim for <135/85 mmHg (aged <80) 145/85 mmHg (aged 80+). Use clinical judgement for people with frailty or multimorbidity	A or C or D	A+C or D A+C+D A+C+D+Spironolactone Treatment. Intensity stratified by CVD risk
JSH, Umemura et al., 2019 [53]	≥140/90 mm Hg (clinic BP).	Aim for <130/80 (<75 years) or <125/75 (high-risk patients). targets for those (aged ≥75 years) are 140/90 and 135/85 mmHg, respectively.	A or C or D as first-line drugs. When a –20/–10 mmHg or greater decrease in BP is targeted, combination therapy should be considered.	Treatment intensity stratified by CVD risk.
ESC/ESH Task Force, 2018 [25]	≥140/90 mm Hg (clinic BP).	Aim for <130/80 mmHg if age <65 years and <140/80 mmHg if age >65 years.	A+C or D (1 pill). Drug treatment may be considered when cardiovascular risk is very high due to established CVD in individuals with BP between 130–139/85–89 mmHg.	A+C+D (1 pill) A+C+D+Spironolactone (2 pills). Treatment intensity stratified by CVD risk
AHA/ACC, Whelton et al., 2017 [26]	≥130/80 mm Hg	Aim for <130/80 mmHg	Single-agent (A or C or D) for BP between 130–140/80–90 mm Hg and high CV risk. Two first line agents (A+ C or D) if BP >140/90 mmHg.	Add more drugs (D or spironolactone as necessary)

ISH = International Society of Hypertension, NICE = The National Institute for Health and Care Excellence, JSH = Japanese Society of Hypertension, ESC = European Society of Cardiology, ESH = European Society of Hypertension, AHA = American Heart Association, ACC = American College of Cardiology, ABPM = Ambulatory Blood Pressure Monitoring, HBPM = Home Blood Pressure Monitoring, BP = Blood Pressure, A = ACE Inhibitor or angiotensin receptor blockers, C = Calcium Channel Blockers, D = Diuretics. In general, ambulatory blood pressure monitoring (ABPM) or home blood pressure monitoring (HBPM) is recommended in ideal settings for diagnosis of hypertension. The ABPM equivalent of clinic blood pressure for diagnosis of hypertension in general is 10 mmHg lower for 24h average or 5 mmHg lower for daytime average or 20 mmHg lower for nighttime average. The HBPM equivalent of clinic blood pressure in general is 5 mmHg lower.

#### New guidelines and recommendations

Several national, regional, and international guidelines and policy documents for the management of hypertension have been published recently (see also Table 2).

**Figure 1:**
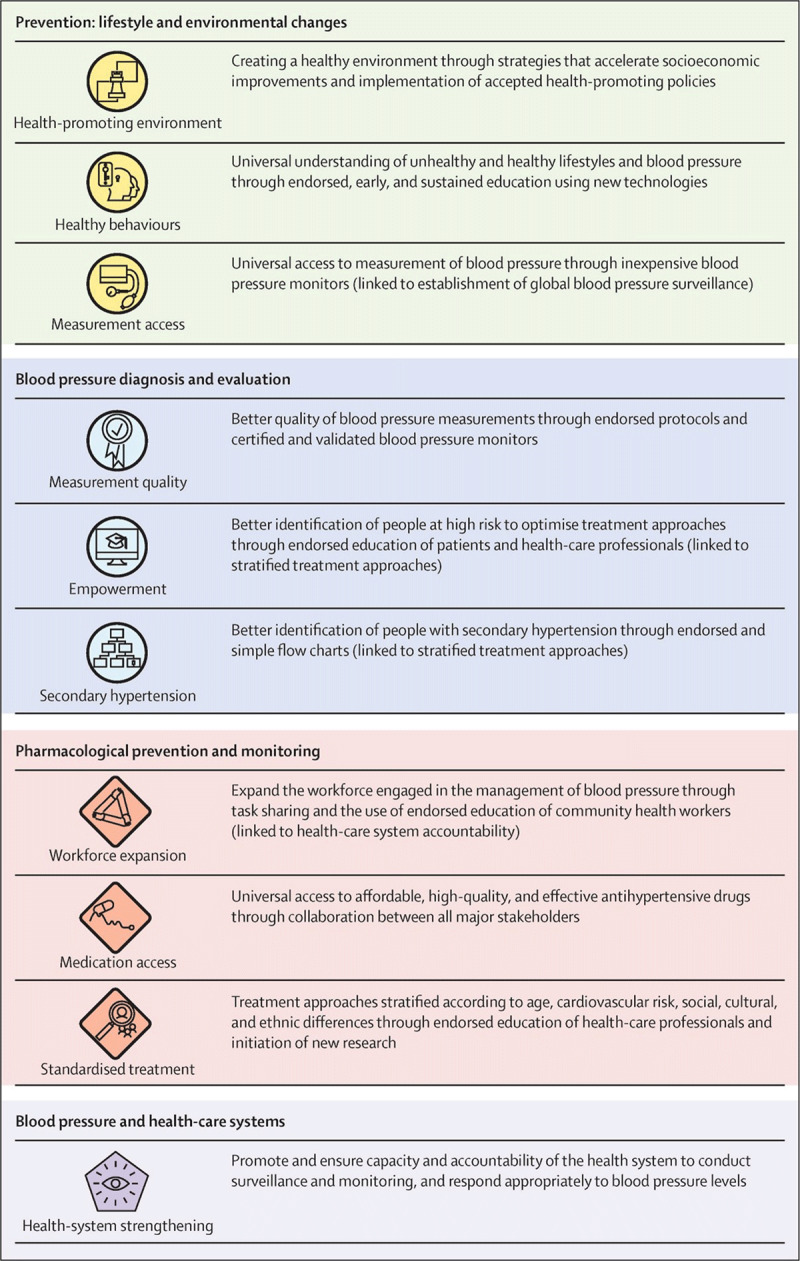
Ten key actions recommended by the Lancet Commission of Hypertension [46]. Reprinted from The Lancet, 388, Olsen MH, Angell SY, Asma S, Boutouyrie P, Burger D, Chirinos JA, et al. A call to action and a lifecourse strategy to address the global burden of raised blood pressure on current and future generations: the Lancet Commission on hypertension., 2665–712, Copyright (2016), with permission from Elsevier.

- In 2016, the Lancet Commission on Hypertension identified 10 key global actions [46] and a strategy across the life-course to address the global burden of hypertension (Figure 1).
- In 2017, new American guidelines for the management of hypertension were released [47].
- In 2017, the Task Force of the Latin American Society of Hypertension released guidelines on the management of arterial hypertension and related comorbidities in Latin America [48].
- In 2018, the European hypertension guidelines were released [25].
- The 2018, the Chinese hypertension guidelines recommended a combined cardiovascular risk and BP level-based antihypertensive treatment algorithm [49].
- In 2018, the evidence-based protocol module of the WHO HEARTS technical package was released and presented an algorithmic approach to managing hypertension in primary care settings. The module consists of a few possible algorithms endorsed by prominent societies including the World Heart Federation, International Society for Hypertension, and the World Hypertension League [50,51].
- In 2019, single-pill anti-hypertensive drug combinations were listed on the WHO Essential Medicine’s list [24].
- Other countries, such as the United Kingdom [52] and Japan [53], also updated and released their guidelines in 2019 (Table 2). In 2020, the International Society of Hypertension [12] released global guidelines targeting essential and optimal standards of care tailored to low- and high-resource settings, respectively.

### New trials and emerging areas of interest

#### Treatment options in ethnically diverse populations

Optimal treatment combinations (required by most patients) are not identified for black, South Asian or patients from East Asia. Whilst the CREOLE trial indicates that the combination of amlodipine with either hydrochlorothiazide (HCTZ) or perindopril is superior to perindopril plus HCTZ in reducing 24-hour BP among black patients from sub-Saharan Africa with hypertension [54], trials with hard outcomes such as cardiovascular morbidity and mortality are required for a clear recommendation for each major ethnic group.

#### Low-dose combination therapy in hypertension management

Low-dose combinations of antihypertensive agents in general are more effective in BP lowering with fewer side effects than high-dose monotherapies [55]. Recent advances in such single-pill combination treatment for hypertension led to the inclusion of four dual combinations of anti-hypertensive agents (lisinopril/amlodipine; lisinopril/hydrochlorothiazide; telmisartan/amlodipine; and telmisartan/hydrochlorothiazide) on the WHO Essential Medicines List. Recent trial evidence suggests that single-pill combinations of two, three and four drugs at lower doses may provide improvements in adherence, efficacy, and tolerability of BP-lowering therapy [56].

#### Intensive blood pressure reduction

The Systolic Blood Pressure Intervention trial (SPRINT) reported significant benefits associated with intensive treatment to achieve a clinic systolic BP target of <120 mmHg [57]. Subsequently, the ACC/AHA guidelines incorporated the findings of the SPRINT trial and revised the definition of hypertension and treatment targets [26]. Given the differences in methods used to measure BP in the SPRINT trial compared with real world situation and other summary data, other guidelines have taken a more conservative approach [12,25,25,53].

#### Fourth-line treatment in resistant hypertension

The PATHWAY 2 trial supports spironolactone as the optimal fourth-line drug (over and above renin angiotensin blockers, calcium channel blockers and diuretics) with an acceptable side effect profile in the treatment of individuals with resistant hypertension [58].

#### Effect of environmental factors on hypertension

Recent research has shown that various environmental factors affect BP and CVD [37,38,39]. For instance, there is significant seasonal variation in BP levels as well the risk of cardiovascular events, with the peak in winter. Data in general show higher BP levels in winter [59,60,61], especially in the elderly [62,63,64,65]. Recently, evidence for the association between air pollution and CVD has been comprehensively reviewed [66], and fine particulate matter <2.5 µg (PM2.5) was identified as a significant contributor to the incidence of hypertension [67]. Asia, Africa, and a few other specific countries where PM2.5 levels are among the highest in the world are particularly affected. Data from Japan showed that the combined effects of low temperature and high PM2.5 concentration substantially increase the BP and the likelihood of morning hypertension [68].

Associations between raised BP levels and low greenness [69,70], poor housing conditions, cooking fuel use [71,72] and indoor air pollution [73] have been observed. Observational studies have also shown that exposures to high air pollution, traffic, noise, and low green space in early life were associated with higher BP in young children [74]. More research into these areas is needed.

## Implementation of the 2015 WHF Roadmap

A key aim of WHF Roadmaps is to provide a framework for regions and countries to develop their own Roadmaps. Over the past few years, the 2015 WHF Roadmap on raised BP has been adapted with the support of WHF in Africa and South America which differ in terms of available resources and context. In Africa, it led to the development of the ‘PASCAR Roadmap on Hypertension: Roadmap to achieve 25% hypertension control in Africa by 2025’ [75], as outlined in **Case Study 1**. The PASCAR initiative resulted in national guidelines, community hypertension programmes, and research projects. In Kenya, this in turn led to a specific implementation programme aimed at accelerating guideline dissemination as illustrated by **Case Study 2**. In early 2020, the Colombian Society of Cardiology and Cardiovascular Surgery (SCCCC) and WHF held a Roundtable in Bogotá, Colombia, using the original WHF Roadmap to help identify obstacles and find solutions at the national level (**Case Study 3**).

From a general perspective, implementation of both the PASCAR and the Colombian Roadmap, similar to the implementation of comprehensive programmes such as HEARTS [76], share the following, common elements:

- A deeper understanding of the culture and health system structure, which have the primary responsibility to manage and fund primary healthcare is essential to scale-up interventions.
- There is a need to continually adapt to the context of each country/setting to account for the fact that implementation does not occur in a linear fashion, but rather as an iterative and dynamic process.
- The paradigm shift to move the gravity centre of CVD/hypertension programmes from specialized secondary care and academic medicine to the community level (primary care) is essential for population-wide impact (see **Case Study 4**).
- Capacity building initiatives to train a new generation of leaders to carry forward the implementation strategy are crucial elements of sustainability and an important characteristic of a resilient health system (see **Case Study 4**).
- There is a strong need for technical leadership to engage, advise, and accompany the process of adoption of interventions at various levels. Cultivating in-country technical leadership is as important as obtaining and sustaining political will.
- A standardized methodology of engagement, pre-implementation, implementation, and institutionalization will improve sustainability of the interventions. A strategy-mapping exercise with all stakeholders is important to understand the setting and ensure the initiative can be sustained within the health system.
- Forming strong alliances with scientific societies, community champions, local level administrators (e.g., the local government authorities) and other academic stakeholders are crucial for impact at the population level.

## Focus areas, barriers, and enablers to scale up recommendations

The major challenge of the integration and scalability of hypertension control initiatives is to translate research findings to real-life settings. However, there is very limited evidence about what to scale up to improve hypertension management in LMICs, and these countries face significant resource constraints, health workforce shortfalls, governance difficulties, lack of international funding, poor governmental prioritization due to competing priorities such as management of malaria, tuberculosis and HIV, maternal and child health, and other challenges in scaling up programmes.

The original WHF Roadmap on raised BP identified a series of roadblocks and solutions on the way to the ideal patient journey. In July and August 2020, we conducted an online consultation of all WHF Members using snowball sampling to include regional members as well as national representatives. In total, we obtained 106 responses from 46 different countries, offering feedback on the roadblocks and solutions identified in the 2014 Roadmap as well as identifying new and emerging barriers and enablers. The data were analysed; open comments were reviewed and consolidated to inform the current updated Roadmap.

Responses received in the online survey showed that most roadblocks identified in 2014 are still relevant particularly in LMICs. In particular, insufficient patient awareness (that they are at risk, that they have raised BP or that they need to adjust to living with their long-term treatment is perceived as a significant roadblock. The lack of availability of locally adapted guidelines, or the fact that healthcare professionals are not aware of guidelines because of a lack of training opportunities, was still identified as a barrier, in particular in LMICs. Respondents from LMICs also identified a lack of understanding of guidelines by healthcare professionals as a barrier, whereas higher-income countries tended to perceive the fact that healthcare professionals do not follow guidelines as an obstacle to optimal care. Likewise, the lack of availability and affordability of priority interventions as well as shortage of healthcare professionals to screen and prescribe were perceived as a roadblock in LMICs, but only marginally in HICs (70% in LICs vs. 20% in HICs, respectively 60% vs. 17% and 92% vs. 30%). Shortage of physicians in rural areas, insufficient primary prevention at primary care level, lack of trained hypertension champions, government-led protocols that do not match patient needs and the proliferation of alternative medicine practitioners and spiritual healers who claim cure for hypertension and mislead the public were identified as additional barriers.

The range of solutions identified in 2014 were also considered relevant in 2020 across country categories. Continuous education for patients and their families, accessible and free hypertension clinics, telemonitoring and use of digital communication between patients and healthcare teams, media campaigns, awareness initiatives targeting young people, physician/pharmacist collaborations, primary care workers empowerment, and systematic delivery of lifestyle recommendations by physicians were identified as additional potential enablers.

Table 3 provides an up-to-date overview of roadblocks and possible solutions based on both WHF members’ feedback and a review of the literature [1,77]. Some of the solutions highlighted in Table 3 are discussed more in-depth in ‘prioritised solutions along the continuum of care for hypertension’ (page 13).

**Table 3:** Roadblocks and solutions.

	Dimension	Barriers	Solutions

**Pre/Diagnosis**	**Demographic and socio-economic factors (individual level)**	Lack of access to testing centres	Facilitate access to health centres where individuals can be diagnosed.
	**Knowledge and beliefs (individual level)**	People are not aware that they are at risk of hypertension/have hypertension. Individuals have a poor understanding of the importance of detecting hypertension.	Implement community awareness campaigns. Roll out opportunistic screening (see case study 2). Implement community- and worksite-based screening and education. Identify and engage local/national champions, including community health workers and volunteers and other non-traditional means to raise awareness [144] (e.g., barber shops[145]). Encourage out-of-office BP measurement (see case study 3). Encourage involvement in and expansion of May Measurement Month.
	**Health systems resources and processes**	Lack of health care professionals to screen/prescribe priority interventions and to provide counselling	Promote task sharing/enhanced scope of practice for non-physician health workers for opportunistic screening and early diagnosis of HT (see case study 5). Provide clinical decision support systems and incentives for health care providers.
	**Social relations, norms, traditions**		
	**Demographic and socio-economic factors (individual level)**	Financial constraints Forgetfulness and poor motivation Competing family and work responsibilities	Support universal health care (UHC) for all and ensure hypertension is adequately covered in UHC coverage plans. Facilitate access to health centres where patients can be followed up free of charge. Provide financial and social support for patients (eliminate user fees and out-of-pocket medication costs). Choose low-cost alternatives in settings where there is idiosyncratic pricing.
**Start of treatment**	**Knowledge and beliefs (individual level)**	Poor understanding of hypertension Doubt that medicine can alleviate symptoms, fear of taking medication Lack of willingness to seek treatment for an asymptomatic condition	Involve families, social networks, local vendors accounting for the fact that many people self-manage using advice from such sources. Support e-health and education of both health care recipients and carers to enable linkage between diagnosis and treatment.
	**Health systems resources and processes**	Health care professionals are not aware of guidelines Health care professionals are aware but do not follow guidelines Lack of understanding of guidelines by healthcare professionals	Educate health care professionals on hypertension risk and guidelines. Implement practical guidelines targeted to LMICs: (ISH guidelines). Promote the HEARTS approach and the ISH 2020 guidelines for use of simple of diagnostic and treatment algorithms (see case study 7). Encourage healthcare workers to share knowledge. Regulate and develop policies to increase the uptake of accuracy validated automated BP devices for routine screening and clinical care (see case study 6).
		Lack of linkage between the diagnosis of hypertension and treatment Lack of staff, medication, and equipment, long queues, long distances Priority interventions are not available Priority interventions are not affordable	Include affordable high-quality long-acting evidence-based and preferably single pill combination generic antihypertensive drugs in the national list of essential medicines. Promote task sharing/enhanced scope of practice for non-physician health workers with prescription rights to trained nurses and pharmacists for first line anti-hypertensive drugs. Ensure that priority interventions are available at the community level (including pharmacies) (see case study 9). Promote local quality-controlled manufacturing, bulk purchasing and/or efficient system to streamline medication supply (see case study 9). Ensure the availability of low-price, good-quality, and resistant sphygmomanometers.
	**Social relations, norms, traditions**	Patient lack of partner and social support Poor relations between health workers and patients Fear of being reprimanded by health workers Poor relationships with family and friends ‘Unhealthy’ social norms and traditions Traditional hierarchical relationships between providers and patients	Involve families, social networks, local vendors. Develop and promote ‘healthy lifestyle’ campaigns (see case study 5). Educate providers and health workers re the need for enhanced communication with patients.
	**Demographic and socio-economic factors (individual level)**	Need to prioritise family, work, domestic commitments	Allow for multi-month medication prescriptions and community medication delivery so that patients with stably controlled BP require less frequent office visits.
**Follow-up and retention**	**Knowledge and beliefs (individual level)**	Patients are not aware of the need for long term treatment and do not understand the care pathway Patients do not adhere to treatment Patients have issues with complex medication regimen, polypharmacy, side effects of medications Beliefs that long-term medication can cause damage to the body	Strengthen patient and carer education. Develop a whole-society approach, including families, media personalities and social networks. Deliver people centred care to include community-based hypertension management and easily accessible and affordable primary health care. Deliver education and campaigns for health care recipients to promote understanding of the importance of long-term treatment. Use information and communication technology to remind and reassure patients about recommendations. Use patient-nominated, non-professional treatment supporters (e.g., spouse, friends, family, peer groups). Strengthen the role of community health workers who often operate across sectors locally. Improve patient experience (e.g., foster interaction with HCWs when dealing with long queues). Utilise interventions with active involvement of patients and patient support groups.
	**Health systems resources and processes**	Same as for ‘start of treatment/drug therapy’	
	**Social relations, norms, traditions**	Same as for ‘start of treatment/drug therapy’	

**Figure 2:**
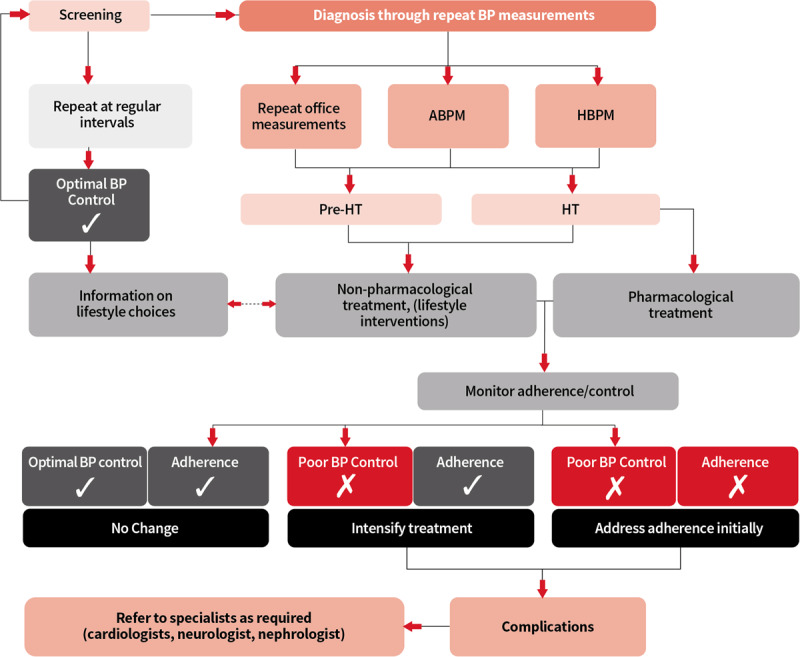
The ideal patient pathway for hypertension, referred to as the Continuum of Care on Page 9 © *World Heart Federation*.

**Figure 3:**
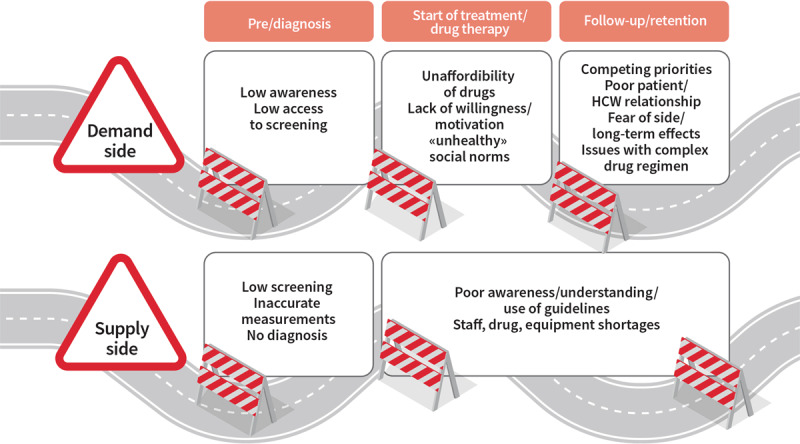
Selected roadblocks on the way to the ideal patient journey © *World Heart Federation*.

The continuum of care for effective management of hypertension covers the domains of prevention, diagnosis, treatment, and long-term care (see Figure 2) and is a useful model for understanding the patient pathways in the management of hypertension [78]. Effective hypertension control involves the whole continuum of care and addressing different roadblocks and enablers at each of the three major stages of the pathway, requiring different approaches: pre-diagnosis/diagnosis, start of treatment, and follow-up/retention in the system. Roadblocks and enablers to hypertension care fall into a range of interlinked domains both at the demand side (e.g., socio-demographic characteristics, health status and co-morbidity, knowledge, and beliefs of hypertension, social relations, and traditions) and supply-side (health system resources and processes) [77]. There are roadblocks and enablers both within and outside the health system – both influence the pathway and retention. These are individual, community- and health system-related, and assume different importance at the major stages of care in the patient pathway [77] (see Figure 3). Contextual relevance and resource availability are two critical elements influencing the sustainability and scalability of hypertension control initiatives in any given setting. It is also important to note that patient pathways are often not linear, which is often not recognised in dominant treatment models [79]. Disruptions in patient pathways and re-entry into any stage is possible based on changes in barriers and facilitators to care at different levels over time. Importantly, barriers and facilitators are interdependent, with patients making trade-offs at different stages based on the context at the time of decision making.

### Some prioritised solutions along the continuum of care for hypertension

Hypertension is a disease of three paradoxes: It is usually easy to diagnose, easy to treat, and easy to control. Yet in many parts of the world it is poorly diagnosed, treated, and controlled. Despite the availability of proven solutions to improve hypertension screening, diagnosis, treatment, and control, the wide-spread use of such strategies is limited, especially in LMIC settings. Cost-effective and patient-centred solutions to address the barriers to care at both the supply and demand side need to be fully implemented and scaled-up to the national and regional level to achieve maximum benefits and achieving better global control rates of hypertension. The implementation science frameworks could be used for tracking the progress and impact of scale-up activities in the management of hypertension [80]. An active surveillance system to monitor progress is an important element in the evaluation of hypertension control initiatives. New initiatives should incorporate effective and sustainable options for improving hypertension management that address the key barriers to optimal hypertension care at different levels of the health system.

Prevention and control of hypertension requires multi-faceted strategies at the individual, community, and population level. At the individual level, it includes efforts towards increased awareness and self-care skills, healthy lifestyle, and access to affordable treatments. At the population level, it includes efforts to generate healthy public policies, healthy living environments, and healthy communities with the aim of addressing the social determinants of hypertension. The following sub-sections discuss some prioritised solutions along the continuum of care for hypertension. Additional possible solutions are listed in Table 3.

#### Develop population-wide prevention and control programmes

Public policies that shape a healthy environment need to be prioritized as they can play a key role in preventing and controlling hypertension. This includes developing and implementing context-specific prevention programmes and raising awareness among policy makers, health professionals, and the general population. Population-wide salt-reduction strategies provide a good example. As an illustration, in a stepped-wedge cluster-randomized trial of a population-wide salt substitution strategy in six villages in Peru, replacing regular salt with a combination of 75% sodium chloride and 25% potassium chloride resulted in 1.3 mmHg population average reduction in systolic BP [81]. Additionally, this salt replacement strategy halved the incidence of hypertension in individuals without hypertension at baseline [81]. As illustrated in **Case Study 5**, other multi-component salt reduction programmes have shown similar benefits.

On a policy level, fostering healthier diets and lifestyles with easy access to healthy foods are established as providing beneficial effects on BP levels (Table 1). In addition, depending on the local context, controlling both indoor and outdoor air pollution, in particular PM2.5 levels, improving greenness levels [70,82] and urban designs, and subsidising cleaner cooking fuels, may also have beneficial effects on BP levels [83].

#### Roll out opportunistic screening

Opportunistic screening for hypertension and frequent measurement of BP in high-risk individuals are important for increasing awareness of hypertension and for early identification of individuals with hypertension and initiation of appropriate treatment options and therefore needs to be scaled up and integrated in hypertension management strategies. However, the availability of BP measurement devices, different types of machines used in the measurement and their measurement related errors cast doubts on the effectiveness of opportunistic screening and further diagnosis of hypertension. In agreement with the WHO quality standards for BP machines, it should firstly remain a priority to have sufficient numbers of machines available to ensure screening and diagnosis, and secondly to ensure that these devices are calibrated and validated appropriately [84].

Real-life examples such as the ‘May Measurement Month’ (MMM), an initiative led by the International Society of Hypertension (ISH) and endorsed by the World Hypertension League (WHL), have shown that it is possible not only to increase the awareness about hypertension but also facilitate BP measurements in large groups of adult participants and detect hypertension or high BP for the first time in a large proportion of them.

MMM, which first took place in May 2017, is an annual globally synchronized BP screening campaign designed primarily to raise awareness of the importance of BP measurement at the population level and in the process, detect untreated or inadequately treated hypertension.

Over 1.2 million people had their BP measured in 2017 with a further 1.5 million people in 2018 and in 2019, arising from over 100 countries from across the world. Screening sites were held in a variety of locations, including hospitals, community centres, schools, supermarkets, and factories. One critical finding of MMM in 2019 was that nearly one-third of the screenees had never had their BP measured previously. Further, in the three campaigns to date [85,86,87], almost one million adults with untreated or inadequately treated hypertension have been detected. These ‘big data’ albeit not based on nationally representative samples can be used where better data are not available to influence policy on screening and management of hypertension.

#### Encourage out-of-office BP measurements (especially home BP monitoring)

Self-home (HBPM) or 24-hour ambulatory BP monitoring (ABPM) is preferred for the diagnosis of hypertension as recommended by the latest hypertension guidelines [12,25,47,48,49]. However, in most countries both HBPM and ABPM are used sparingly in primary care settings for the diagnosis or management of hypertension. Effective and sustainable models for implementing out-of-office BP testing need to be developed to strengthen the scale up of hypertension control initiatives. HBPM is generally preferred because ABPM is more expensive, less available, and less popular with patients particularly for repeat use.

Out-of-office self-measurement or 24-hour ambulatory monitoring are not practical in many LMIC settings due to resource limitations. However, non-physician health worker-led monitoring of BP at the household level at frequent intervals may be an alternative strategy to diagnose hypertension and tailor treatment options, provided the quality and accuracy of the measurement can be guaranteed (see **Case Study 6**).

#### Strengthen primary care

Primary care physicians and other allied healthcare workers should be in the forefront of the management of hypertension. Their competencies in providing evidence-based management of hypertension are key to achieving optimal BP control rates at the population level. Several large-scale initiatives, which rely strongly on primary care, such as Global HEARTS and Resolve to Save Lives, were launched in the past five years to support governments and health authorities in strengthening CVD prevention and control. A priority should now be to learn from these programmes to scale up their implementation in relevant settings (see also **Case Study 7**).

#### Promote and implement task sharing and team-based care

Wagner’s Chronic Care Model [88] aims to prioritise and facilitate engagement and ownership by stakeholders through a process mapping exercise. This may help identify health system barriers including clinician-based factors [89] to allow effective delivery of optimal hypertension care. Team-based care has been identified and recognised as an important strategy to improve hypertension control in multiple settings [90,91,92].

Similarly, task sharing strategies that involve transferring less skilled tasks to non-physician health workers under the supervision of a physician if required are effective in addressing some of the key barriers both at the demand and supply side in the scale up of interventions to improve hypertension control. A recent systematic review and meta-analyses support task-sharing interventions of involving multiple cadres of non-physician health workers (community health workers, medical assistants, nurses, dieticians, and pharmacists) in the management of hypertension in LMIC settings [93]. Strengthening the implementation of task-sharing and team-based care is therefore a priority for the near future in these settings.

As an example, the COBRA-BPS study in rural settings of South Asia provides convincing evidence of a community health worker-led multi-component intervention strategy for BP control [94]. This intervention is scalable to national level in low-resource settings. It includes regular home visits from trained community health workers who monitored BP and provided health education and counselling. In addition, local physicians were trained to follow a simple treatment algorithm to provide medications. A strong component of care coordination across different cadres of health workers and patients were part of the COBRA-BPS model. This model resulted in a greater reduction in BP in comparison to usual care [94]. Similarly, a nurse-led task-sharing intervention strategy resulted in a greater reduction in systolic BP in a cluster randomised controlled trial in 32 communities in Ghana [95]. Other community health worker-led programmes have shown similar results [96,97]. However, multiple barriers prevent utilising non-physician health workers in the management of hypertension and will have to be overcome [98]. As an example, and already in practice in some countries, legal frameworks should be adapted so that non-physician health workers are duly authorized to prescribe first line treatment of antihypertensive medication. Conducting more research in this area should also be considered.

#### Deliver people-centred care

Achieving optimal hypertension care requires the implementation of interventions on scale. However, increasing the scale and maximising the reach of interventions are influenced by several factors such as the implementers, potential end-users, choice of a delivery strategy, and the socio-political and economic contexts. Shifting the attention to a co-production model where patients and families have an important role in decision making helps to scale-up intervention strategies for the control of hypertension [77]. A broader people-centred model (envisaging roles not only for patients but also for families/networks/society) is a possible way to improve adherence to self-care and therapeutic options in the management of hypertension [99].

#### Strengthen patient and carer education

Communication gaps between patients and providers [100], poor clinical handover or patients discontinuing medications for hypertension due to inadequate awareness of the importance of lifelong medications [101,102] are well-documented barriers to optimal care for chronic conditions in low resource settings. Patient and carer education is therefore perceived as an important strategy to improve hypertension management in primary care settings [103]. Education needs to be patient-centred rather than provider-centric, for example by creating simple infographics and picture-based educational materials for low literacy groups. Meanwhile, carer education is necessary to ensure that guidelines are understood and implemented and that good communication with patients is critical. Patient and carer education strategies therefore both need to be incorporated as part of scale-up interventions in the management of hypertension.

#### Facilitate adherence to pharmacological therapy

Despite the benefits of persistence with anti-hypertensive treatments [104], adherence to key therapies are often low in individuals with hypertension. Non-adherence to anti-hypertensive treatment occurs when a patient does not initiate a new prescription, up titrate the drugs as prescribed, or continue with treatment [105]. The complexity of dosages, requirements for multiple pills, inadequate patient training, side-effects to high dose single drugs, and cost of treatment often affect adherence to anti-hypertensive treatment. The latest treatment guidelines give preference to once-daily single-pill combination therapy with effective and well-tolerated agents [12,25] to reduce pill burden, simplify treatment regimens, and improve treatment adherence in hypertension. A priority will be to foster their dissemination and implementation. Relatively long-term prescriptions for management of hypertension and refill of medicines once in three months should be considered whenever possible. It may remove the requirement for frequent travel to a health care facility, a major barrier to treatment adherence. Where possible the use of a reminder electronic pill box and monthly pill counts are used as strategies to improve or evaluate medication adherence in individuals with hypertension [106]. However, a confirmed record of collection of medicines does not ensure use of the medications. Further, electronic medical event monitoring systems, electronic pill boxes and biomedical testing are costly, and often not practical in resource poor settings. Other dose administration aids (e.g., pharmacist-packed blister boxes, self-packed reminder boxes, or machine-packed sachets) may also be cost-effective and improve adherence and BP control. From a health-system perspective, reducing or eliminating patient out-of-pocket costs for hypertension services and promoting global transparency and equity of antihypertensive medication prices are key elements that facilitate adherence to treatment.

#### Improve medication supply management

Inadequate supplies necessary for hypertension management are a major barrier to the delivery of optimal care for the management of hypertension in low-resource settings [107].

There is therefore a need to engage the health system to ensure an adequate supply of essential anti-hypertensive drugs in primary care settings. Relying on simple treatment protocols with specific drugs and doses at each step is recommended to facilitate the procurement of large volumes of medicines of choice, which simplifies inventory and supply chain, and lowers prices (see also **Case Study 8**).

#### Foster the use of novel technologies (DSS, m-health, e-health, apps, etc)

Decision support systems (DSS) help health care providers adhere to hypertension treatment guidelines. Further, DSS maintain electronic health records for ongoing monitoring and facilitate clinical handover. Hypertension control initiatives based on DSS are effective in improving BP control in individuals with hypertension [108]. Further, they also facilitate task-sharing interventions of involving nurses and pharmacists in the management of hypertension.

mHealth, which WHO defines as ‘the use of mobile and wireless technologies to support the achievement of health objectives’ [109], also offers a wide range of possibilities, for example for patient and carer education, patient support, as well as adherence to treatment and lifestyle recommendations – reviews have already shown that mHealth interventions can contribute to improving medication adherence and BP control [110,111]. For example, in the HOPE 4 study, a community-based cluster randomised trial in 30 communities, an mHealth-enabled non-physician health worker-led intervention improved BP control and reduced cardiovascular risk substantially in individuals with hypertension [112]. One of the strengths of mHealth interventions is their scalability. A priority for the coming years must therefore be to leverage their potential to improve the management and control of hypertension, whilst keeping in mind that to be effective, such interventions need to be carefully selected, planned, rolled out, and evaluated. When implementing novel technologies, a particular focus must lie on issues pertaining to interoperability as well as data protection and security.

### Conclusions and recommendations

Cost-effective lifestyle and medical interventions to prevent and manage hypertension do exist. However, uptake of both is still low, particularly in resource-poor areas. This negatively affects progress towards achieving the SDG target 3.4 of ‘one-third reduction in premature mortality from non-communicable diseases (NCDs) by 2030’. This Roadmap examines the roadblocks pertaining to both the demand side (demographic and socio-economic factors, knowledge and beliefs, social relations, norms, and traditions) and the supply side (health systems resources and processes) along the patient pathway. It proposes a range of possible solutions to overcome them. The ideal journey includes a range of commitments/achievements on both the supply and the demand side, as illustrated in Table 4.

**Table 4:** Commitments/achievements on the supply and the demand side.

Supply	Demand

Governmental and societal willingness to make hypertension control a priority	Individual awareness of own BP
Shaping healthy environments to facilitate the choices of individuals towards healthier lifestyles	Individual lifestyle modifications
Treatment with cost-effective – and affordable – medications	Adherence to treatment
Education of both HCPs and patients to address awareness, facilitate and encourage adherence to treatment and understanding that BP control is a lifelong commitment.	

Barriers and solutions differ according to region and should be tailored to each setting, taking into account the needs and circumstances of the users and their community, within a people-centred model of hypertension management. This Roadmap update has looked at possible solutions based on new and emerging fields of interests that now need to be scaled up to improve hypertension prevention, detection, and control around the world.

1. Develop population-wide prevention and control programmes
2. Roll out opportunistic screening
3. Encourage out-of-office BP measurements (especially home BP monitoring)
4. Strengthen primary care
5. Promote and implement task-sharing and team-based care
6. Deliver people-centred care
7. Strengthen patient and carer education
8. Facilitate adherence to pharmacological therapy
9. Improve medication supply management for example by including affordable high-quality long-acting evidence-based and preferably single pill combination generic antihypertensive drugs in national lists of essential medicines
10. Foster the use of novel technologies (m-health, e-health, apps, etc).

**Case Study 1: PASCAR Roadmap**

**Context**

Given the high burden of hypertension in Africa, the Pan African Society of Cardiology (PASCAR), through its hypertension task force, decided to prioritize the control of hypertension in Africa. At a meeting in Nairobi, Kenya, in 2014, it was decided to develop a Roadmap for the control of hypertension in Africa following the framework of the recent WHF Roadmap.

**Process**

**Figure 4:**
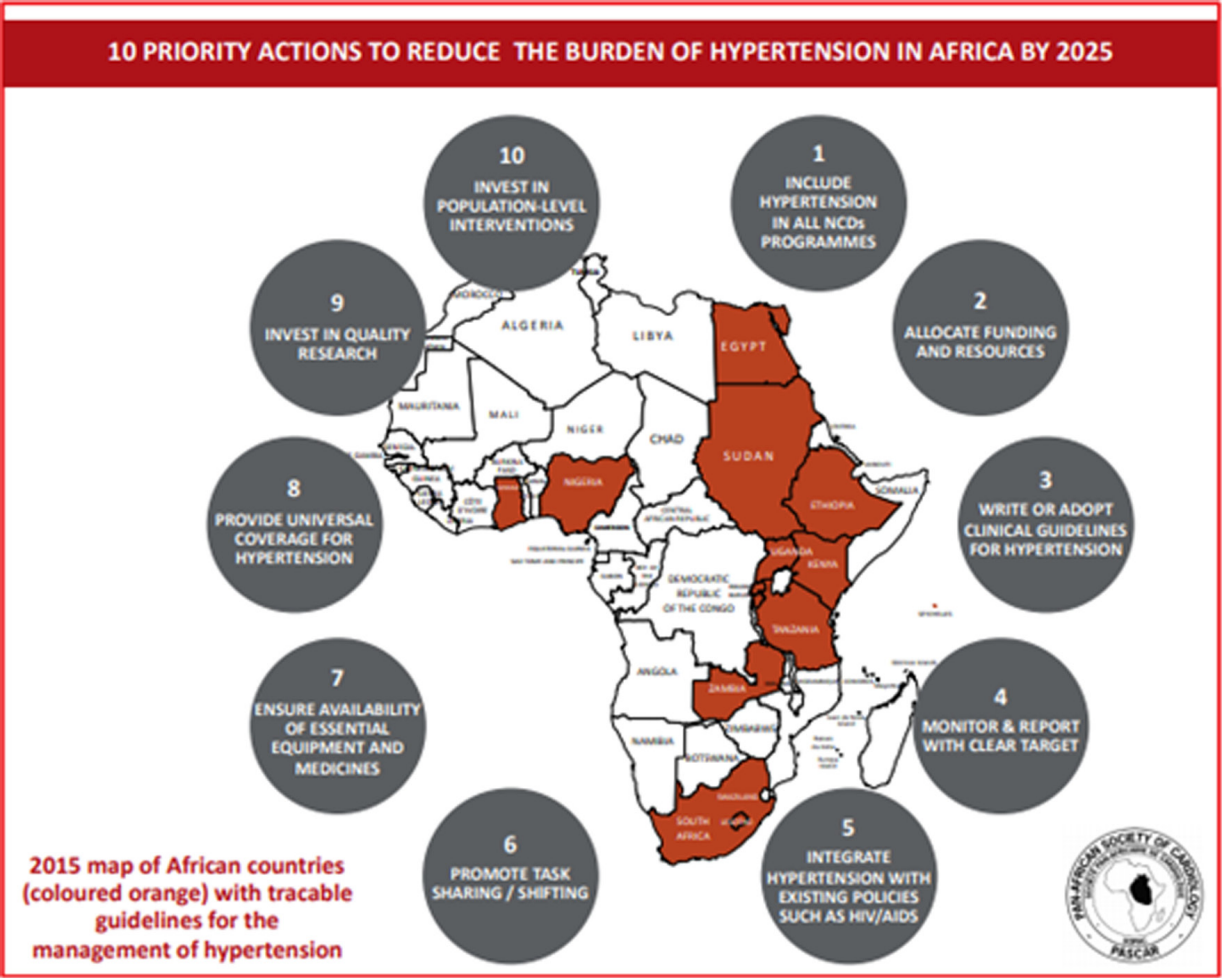
PASCAR Roadmap on Hypertension with a 10-point action plan. *Reprinted from Global Heart, 13(1), Dzudie A, Rayner B, Ojji D, Schutte AE, Twagiramukiza M, Damasceno A et al, Roadmap to Achieve 25% Hypertension Control in Africa by 2025, pp. 45–59, 2018, with permission from Ubiquity Press*.


The PASCAR created a Taskforce, which included several African countries to develop a local framework to prioritise the control of hypertension in Africa. A comprehensive review of the literature on hypertension in Africa showed that only one in four countries had clinical practice guidelines on the management of hypertension. The Taskforce identified barriers (roadblocks) in three distinct domains: government and health system; healthcare workers; and patients. Further, the Taskforce discussed solutions to these barriers in detail within the African context and prepared a draft Roadmap. The draft was reviewed by experts from organisations such as ISH, WHF, and national cardiac societies. Ultimately, the ‘PASCAR Roadmap on Hypertension: Roadmap to achieve 25% hypertension control in Africa by 2025’ [75] with a 10-point action plan (Figure 4) was published simultaneously in the *Cardiovascular Journal of Africa* and *Global Heart* in October 2017.

**Implementation plan**

The national cardiac/hypertension societies were in liaison with the relevant ministries and stakeholders primarily responsible for implementing the Roadmap. Additionally, the Taskforce sought collaborations with WHO-AFRO, WHO-EMRO, and the African Union Council of Ministers of health for the successful implementation of the Roadmap.

**Outcomes**

The PASCAR initiative resulted in the production of national guidelines, community hypertension programmes such as Health Heart Africa (HHA) in Kenya [113,114], and research projects such as the CREOLE trial [54].

**Case Study 2: Kenya cardiovascular guidelines dissemination**

**Figure 5:**
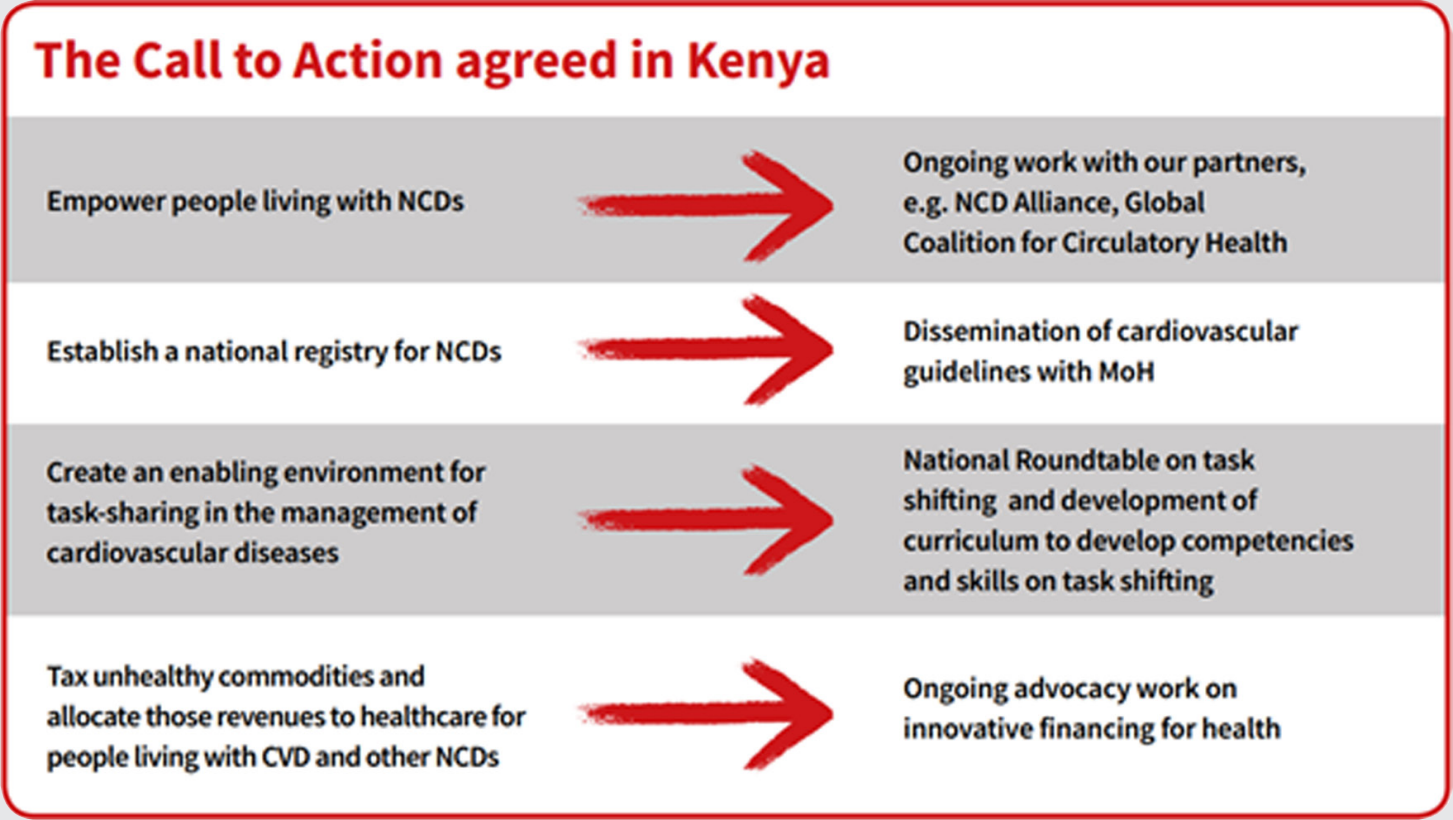
The Call to Action agreed in Kenya, reprinted from ‘Accelerating Cardiovascular Health and Care in Kenya’, available at https://www.world-heart-federation.org/wp-content/uploads/2019/01/WHF-Care-in-Kenya-brochure_WEB.pdf, with permission from WHF.

In March 2018, the WHF and the Kenya Cardiac Society (KCS) convened a Roundtable meeting entitled *Accelerating Solutions to Hypertension Management*, which brought together representatives from the Ministry of Health and country health directorates, primary health care, civil society, the private sector, academia, and faith-based organisations. A call to action was agreed, as shown in Figure 5.

In addition, the stakeholders prioritised the dissemination of the guidelines recently published by the KCS in conjunction with the Ministry of Health. The process was staged between March 2019 and July 2020. It included a one-day national stakeholder workshop, centralised training of trainers as well as two one-day training sessions per county for healthcare professionals. As a result, five counties, 25 trainers, 100 facilities were reached, and 277 healthcare professionals were trained. The dissemination phase is undergoing evaluation, and its extension to six additional counties is under planning.

**Case Study 3: A national Roadmap for Colombia**

The WHF, together with the Colombian Society of Cardiology and Cardiovascular Surgery (SCCCC), held a Roundtable in Bogotá, Colombia in January 2020 based on the original WHF Roadmap to identify obstacles and find solutions to them at the national level.

To advance efforts towards the control and management of hypertension in Colombia, WHF and the SCCCC identified a series of locally relevant roadblocks:

*The health system lacks data on incidence and prevalence of hypertension in Colombia*.

*BP measurement is not standardized.*

*The population lacks awareness of hypertension.*

*The population is not sufficiently aware of the risks caused by sodium-rich foods.*

*Patients are unaware that they are at risk of hypertension/unaware of the hypertension status.*

*Health technologies are not sufficiently accessible*.

A series of strategies and solutions were then delineated based on the previously identified roadblocks. Those are yet to be implemented. As a first step, based on the observation that an efficient hypertension control programme cannot be optimal without an adequate BP measurement system, an extensive campaign was launched in August 2020, to (1) increase the proportion of people who know their BP readings, (2) certify health professionals, such as physical therapists, nutritionists, respiratory therapists, physical educators, dentists, and caregivers in BP measurement through a free online course, and (3) offer lifestyle recommendations to prevent and control hypertension.

**Case Study 4: Capacity-building initiative to scale up evidence-based management of hypertension in primary care**

A capacity-building initiative to enhance the knowledge, skills, and core competencies of primary care physicians in prevention and management of hypertension was conceived, developed, introduced, and evaluated by the Public Health Foundation of India (PHFI) in collaboration with the British & Irish Hypertension Society (BIHS), the International Society of Hypertension (ISH), and the Centre for Chronic Disease Control (CCDC) [115]. The initiative named as the Certificate Course in Management of Hypertension (CCMH) was introduced initially in India as a nationwide program. The CCMH is an eight-month on-the-job training programme with a focus on learning the key principles and evidence-based treatment options in the prevention and control of hypertension. Subsequent to the initial launch and acceptance of this programme among primary care physicians and policy makers, it has been adopted as a model by several state governments in India (Madhya Pradesh, Meghalaya, Manipur, Odisha and Tripura, and the Municipal Corporation of Kolkata) and in worksite settings. The programme has so far trained over 2000 primary care physicians in India. The PASCAR is planning to deliver the course in over 50 countries of the African region to approximately 25,000 prescribers and 50,000 non-prescribers by the year 2025 [116]. The CCMH has been also expanded to countries like Afghanistan and Nepal. Currently, PHFI is developing an online version of this capacity building initiative in collaboration with the American Heart Association. Once available, measurable outcomes will contribute to reinforce the validity of the programme.

**Case Study 5: Successful sodium intake reduction programmes in Asia**

High dietary sodium is a leading contributor to hypertension. It causes 3 million deaths each year, 80% of which occur in low- and middle-income countries, and nearly 50% among people younger than 70 years. Globally, average salt intake is almost twice the recommended level. In 181 of 187 countries, covering 99% of the world’s population, estimated average levels of salt intake exceed WHO’s recommendation of 5 grams per day [117]. In 2017, WHO designated ‘sodium intake reduction’ as a ‘best buy’ to avoid premature deaths and reduce the economic impact of NCDs in LMICs [118].

Most programmes that have been successful in reducing dietary sodium intake include multi-component strategies. For example, the Japanese Society of Hypertension (JSH) set up a Salt Reduction Committee in 2005 to promote the reduction of population salt consumption. In 2019, the JSH has announced the ‘Tokyo Declaration in Promotion of Salt Reduction by the Japanese Society of Hypertension-the JSH Tokyo declaration’ [119]. The action plan included six strategies to achieve the target salt intake level of <6 g/day: (1) educate the citizens on the harms of excessive salt intake and the importance of reducing salt intake; (2) recommend the assessment of salt intake of individuals or population, and propose adequate methods to reduce salt intake; (3) promote salt reduction in children as a part of dietary education at school; (4) promote salt reduction in eating out, prepared dishes, canteens, and school lunches; (5) encourage companies to develop and spread low-salt foods; and (6) encourage the government to take measures to promote salt reduction. Based on the National Health and Nutrition Survey of Japan, salt intake gradually decreased from 12.4 g/day in 2005 to 11.0 g/day in 2018 for men and from 10.7 g/day to 9.3 g/day for women, respectively. Along with the decrease in salt intake, the number of hypertensive patients who take antihypertensive drugs increased during this period, resulting in an overall decrease in blood pressure. As an example, in 2016 the treatment rate of hypertensive men in their seventies increased from 53.5% in 2000 to 66.9% and the average systolic BP of the corresponding population decreased from 147.1 mmHg in 2000 to 140.2 mmHg [53]. In parallel with the improvement in BP control, a 17.5 % decrease in stroke death from 132,529 in 2000 to 109,320 in 2016 was observed [120].

**Case Study 6: Blood pressure measuring devices – Building a case for a robust regulatory framework**

Improved capacity for accurate BP measurement is a prerequisite for better management and control of hypertension. However, from around 3000 cuff-based BP measuring devices (BPMDs), less than 15% have published data on accuracy performance [121]. In addition, new technologies using bio-sensors and cuff-less methods have been emerging in the recent past. However, due to the lack of stringent regulatory frameworks on accuracy standards, many BPMDs on the market that are used both in clinical practice and for home monitoring are likely to be inaccurate [121,122]. In 2020, WHO updated its ‘Technical specifications for automated non-invasive BPMDs with cuff’ [123]. In this report, WHO calls upon governments and health authorities to ‘strengthen their regulatory capacity to ensure that only certified, accurate, and validated BPMD are marketed and to identify institutions in which independent validation can be conducted’. Manufacturers are called upon to submit electronic BPMD for independent validation testing with a rigorous international protocol (e.g., ISO 81060–2; 2018).

Further, healthcare institutions and personnel are called upon to use only validated automatic BMPDs and to undergo adequate training [123]. Importantly, validated BPMDs are now affordable, at least in some parts of the world. The HEARTS in the Americas initiative is also promoting a coordinated effort to accelerate the global adoption of an appropriate regulatory framework that guarantees accurate BP measurement, with validated automatic BP measuring devices (BPMDs[122]). In partnership with the World Hypertension League (WHL) and the Lancet Commission on Hypertension and others, a list of resources for advocacy and country support has been promoted (see www.stridebp.org, which is affiliated with the ESH, the ISH and the WHL) and an online certification course in BP measurement launched [121,124,125,126]. In the future, a robust regulatory framework will be essential to ensure the reliability of BP measurements, performed both in clinical settings and at home.

**Case Study 7: Global HEARTS, Resolve to Save Lives and other HTN control programmes in a nutshell**

Global HEARTS, the WHO’s flagship initiative for CVD prevention and control, was launched in 2016 in partnership with the United States Center for Disease Control and Prevention (US CDC). The initiative comprises five technical packages, four focused on prevention (MPOWER, SHAKE, ACTIVE, and REPLACE) and one focussed on management in primary care – the HEARTS technical package. It encompasses healthy lifestyle counselling, evidence-based protocols, access to essential medicines and technologies, risk-based CVD management, team-based care, and systems for monitoring hypertension and other chronic NCDs [20]. HEARTS provides proven, affordable, and scalable solutions to improve control of hypertension at the primary care level. Globally, this initiative has led to 3 million people receiving treatment for hypertension [21].

The HEARTS in the Americas initiative, led by the Pan American Health Organization (PAHO) with other partners, is a good example of the regional approach to Global HEARTS. This serves as a demonstration model for countries on how to scale up national primary health care-based CVD management programs, including hypertension and secondary CVD prevention, in the region by 2025 [127,128,129]. As a regional convener and technical advisor, PAHO has been able to assure a standard approach to programme design, trainings, implementation, and evaluation across all countries. The programme has so far been implemented in 12 countries (Barbados, Colombia, Chile, Cuba, Argentina, Ecuador, Panama, Trinidad and Tobago, Dominican Republic, Mexico, Peru, and Sant Lucia), covering 371 health centres and 6 million adults.

Tangible results have already been observed in Latin America and the Caribbean. While over 50 medications and more than 100 preparations were used to treat hypertension in the primary care setting at the inception of the programme, HEARTS is now moving towards a formulary that consists of 7–8 core, high-quality medications with 19–20 preparations for use in primary care. In the future, this shift toward preferred antihypertensive medications has the potential to drive down medication prices across the region. In addition, the program successfully improved anti-hypertension formularies and established national pharmacologic treatment protocols [51]. The programme has already led to rapid and significant increases in hypertension control rates [130]. For instance, in the city of Matanzas, Cuba, where HEARTS has been implemented, coverage increased from 52.9 percent to 88.2 percent, and the proportion of those treated who were controlled rose from 59.3 percent to 68.5 percent [130] (see also case study 8).

Resolve to Save Lives (RTSL), a large-scale initiative of the global public health organization Vital Strategies, supports Hearts in the Americas and national hypertension control programmes in ten low- and middle-income countries outside the Americas region to adapt and implement the HEARTS technical package. In addition, it provides funding, technical, and programmatic support to national and local partners to help scale up proven strategies to improve control of high BP, including a) simple and established treatment protocols, b) reliable, affordable supply of quality-assured medications, b) team-based care, c) patient-centred, community-based care, and d) longitudinal hypertension registries to support performance reporting and continuous quality improvement. As of April 2021, RTSL-supported national hypertension programmes had enrolled about 1.8 million hypertension patients in the 10 countries they support outside of the Latin American and Caribbean region.

**Case Study 8** describes the India Hypertension Control Initiative, a collaboration between the Indian Council for Medical Research, the WHO, and RTSL.

*N.b.: These programmes are complementary to the WHF Roadmap initiative. Likewise, they complement and reinforce each other. Synergies between them are possible and encouraged*.

As illustrated in Table 5, other programmes in different regions and settings share a range of common components, demonstrating that organised and sustained efforts can lead to significant improvements in hypertension detection, management, and control over time.

**Table 5:** Successful features of HT control programmes.

Programme	Country	Year started	Key components	Control rate

HEARTS Cuba [130]	Cuba	2016	Highly organized, comprehensive, accessible primary care system Affordable medications Education and training for the public and patients to improve awareness and self-management Standardized training for healthcare professionals Simple directive diagnosis and treatment algorithm Registry providing performance reports Dedicated funding	From 37.7% to 58% in 1 year (overall population)
HOPE 4 [112]	Colombia and Malaysia	2014	Community screening Treatment of risk factors by non-physician health workers using management algorithms Counselling programmes Free antihypertensive and statin medications Support from family or friend	69% (intervention group) 30% (control group)
Yaroslavl programme [146]	Russia	2011	Specific training for healthcare professionals Public awareness program Patient registry with performance reporting Patient recall system	17% to 33% in 4 yrs (overall population)
Kaiser Permanente Northern California Program [147]	USA	2004	Treatment algorithm Regularly updated hypertension guidelines Team-based care, BP measurements by medical assistants Registry with performance reports Single-pill combination therapy Quality performance metrics	From 44% to 90% in 13 yrs (clinical population)
Canadian Hypertension Control Program [130,148]	Canada	Start mid-1990s	Regularly updated management recommendations Standardized education to primary care Education for the public and patients Dedicated leadership position	13% to 66% in 6 yrs (overall population)

**Case Study 8: India Hypertension Control Initiative (IHCI)**

**Figure 6:**
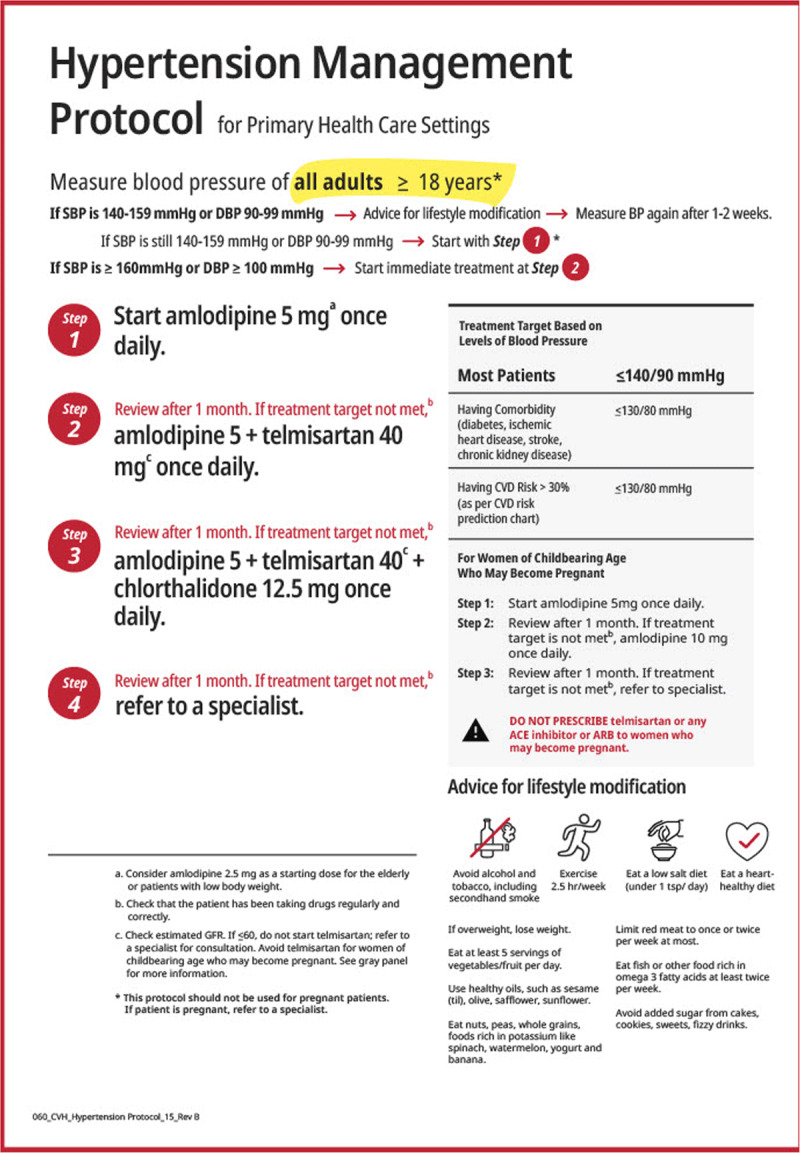
Hypertension Management Protocol for Primary Health Care Settings, *reproduced with permission from Resolve to Save Lives*.

The India Council of Medical Research, the WHO, Resolve to Save Lives, and national and state government stakeholders collaborated to launch the IHCI. The IHCI started in selected districts within five Indian states (Punjab, Maharashtra, Madhya Pradesh, Kerala, and Telangana). Consensus conferences were held in each state, resulting in state-specific adaptation of hypertension treatment protocols based on HEARTS as shown in Figure 6. Validated, automated oscillometric BP monitors were provided to health care facilities. Health worker training and clinical supervision and quality improvement activities were led by IHCI partners and each district’s Cardiovascular Health Officer appointed under the project. Ongoing efforts worked to improve medication supply forecasting, procurement, and supply chain management and the quality of information gathering, BP measurement, and hypertension treatment. During 2020–2021, an expansion added additional districts within these states and selected districts within the remaining states of India. As of the end of April 2021, the IHCI had enrolled over 1.2 million hypertension patients.

However, the lack of detailed data on programme coverage especially in vulnerable groups, the need to improve treatment follow-up and hypertension control rates, and the need to improve medication supply chain and inventory management are crucial obstacles to overcome before recommending scale up of similar strategies in other LMICs.
